# Occupation- and age-associated risk of SARS-CoV-2 test positivity, the Netherlands, June to October 2020

**DOI:** 10.2807/1560-7917.ES.2020.25.50.2001884

**Published:** 2020-12-17

**Authors:** Brechje de Gier, Priscila de Oliveira Bressane Lima, Rolina D van Gaalen, Pieter T de Boer, Jeroen Alblas, Marc Ruijten, Arianne B van Gageldonk-Lafeber, Toos Waegemaekers, Anja Schreijer, Susan van den Hof, Susan JM Hahné

**Affiliations:** 1 Centre for Infectious Disease Control, National Institute for Public Health and the Environment (RIVM), Bilthoven, the Netherlands; 2 GGDGHOR Nederland, Utrecht, the Netherlands; 3 Public Health Service Gelderland Midden, Arnhem, the Netherlands; 4 National Consultation on Infectious Disease Control (LOI), Public Health Service (GGD) Amsterdam, the Netherlands

**Keywords:** SARS-CoV-2, occupation, COVID-19, Netherlands

## Abstract

High coronavirus incidence has prompted the Netherlands to implement a second lockdown. To elucidate the epidemic’s development preceding this second wave, we analysed weekly test positivity in public test locations by population subgroup between 1 June and 17 October 2020. Hospitality and public transport workers, driving instructors, hairdressers and aestheticians had higher test positivity compared with a reference group of individuals without a close-contact occupation. Workers in childcare, education and healthcare showed lower test positivity.

An understanding of the main factors contributing to community transmission of coronavirus disease (COVID-19) is urgently needed to inform targeted prevention policies so that further transmission can be controlled while minimising effects on society and the economy. Here we present test positivity by age group (for individuals < 25 years old) or by occupational group (≥25 years old), per week, from June to October 2020 in the Netherlands.

## Epidemiological situation and mass testing

**Figure 1:**
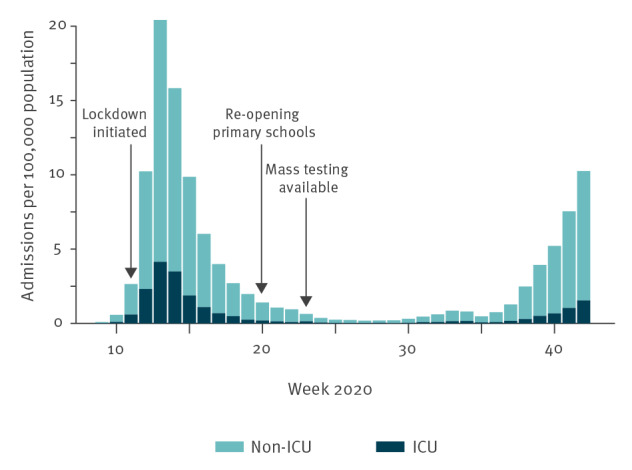
Weekly number of new COVID-19 hospitalisations and ICU admissions^a^, the Netherlands, March–October 2020

COVID-19: coronavirus disease; ICU: intensive care unit; NICE: National Intensive Care Evaluation.

^a^ As registered by Stichting NICE [10].

The first case of COVID-19 in the Netherlands was diagnosed on 27 February 2020, after which incidence increased rapidly, peaking at 20 new COVID-19 hospitalisations per 100,000 population in week 13 (22–28 March). A nationwide lockdown was implemented on 12 March, resulting in a rapid decline in the number of COVID-19–related hospitalisations (Figure 1). The number of hospitalisations plateaued after week 23 (1 June), with 0.5 weekly hospitalisations per 100,000 population. Lockdown measures were gradually phased out in mid-May; however, the incidence of laboratory-confirmed COVID-19 hospitalisations started to slowly increase again over the course of summer. As at week 42 (mid-October), weekly hospitalisations had increased to 10 per 100,000 population.

Since 1 June 2020, the Netherlands has made severe acute respiratory syndrome coronavirus 2 (SARS-CoV-2) PCR testing available for anyone experiencing symptoms compatible with COVID-19 (fever, cough, sore throat, shortness of breath, myalgia, runny nose, sudden loss of smell or taste), as well as for source and contact tracing. Individuals can apply for a test through a national call centre or, since 12 August, via an online portal. The 25 regional Public Health Services (PHS) perform sampling in public test locations. Demographic data, dates of testing and laboratory results are entered into a dedicated IT system named CoronIT. Test results are exported from CoronIT into the HPzone software at the PHS, wherein source and contact tracing data are registered. The National Institute for Public Health and the Environment (RIVM) accesses the anonymised data in CoronIT for surveillance purposes.

When requesting a test, either online or through the call centre, individuals are asked about their occupation. The question is posed as: “*Have you worked in the past two weeks in the capacity of*:”, followed by a list of employment categories potentially associated with SARS-CoV-2 infection risk (Table 1). If a patient reports having recently worked in healthcare or in a close-contact profession, i.e. a profession requiring contact with other persons within 1.5 m, a follow-up question is triggered that asks the respondent to select a specific healthcare or close-contact profession.

**Table 1:** Occupational categories and subcategories registered in CoronIT, occupation- and age-associated risk of SARS-CoV-2 test positivity study, the Netherlands, 2020

Occupational category	Occupational subcategory
Healthcare worker or paramedic (hospital, long-term care facility or elsewhere)	Physician
	Audiologist
	Dietician
	Physiotherapist, occupational therapist, remedial therapist, podiatrist
	(Clinical) aesthetician
	Speech therapist
	Optometrist
	Dentist, dental hygienist
	Nurse
	Caregiver
Close-contact^a^ professional	Hairdresser
	Aesthetician
	Manicurist
	Pedicurist
	Driving Instructor
	Sports trainer or instructor
	Retail employee
	Other
Daycare personnel (for children aged 0–3 years)	–
Elementary school or after-school care personnel (for children aged 4–12 years)	–
Secondary school personnel (for children aged ≥ 12 years)	–
Higher education personnel (for those aged ≥ 16 years)	–
Hospitality^b^ worker with client contact	–
Public transport worker with client contact	–
Informal caregiver	–
Police, military police, firefighter, corrections officer	–
Other occupation not involving close contact^a^	–

SARS-CoV-2: severe acute respiratory syndrome coronavirus 2.

^a^ Defined as requiring contact with other persons within 1.5 m.

^b^ Defined as hotels, restaurants, cafes and bars.

In the Netherlands, 25 regional Public Health Services perform sampling in public test locations. Demographic data, dates of testing and laboratory results are entered into a dedicated IT system, named CoronIT.

Using the numbers of negative and positive tests, binomial proportions of positive tests and accompanying 95% confidence intervals (CIs) were estimated by week of sampling and stratified by occupational category or age group for persons ≤ 24 years of age, in R 3.6.0 (prop.test, package ‘stats’) [1]. Individuals ≥ 25 years that reported working in a non-close-contact occupation were used as a reference group. Healthcare workers (HCWs) were stratified by the setting in which they work (hospital, long-term care facility, elsewhere). Tests registered as performed in the context of source and contact tracing were excluded from all analyses. Total number of tests and number positive per category can be found in Supplementary Table S1. We defined relevant differences as non-overlapping 95% CIs between the weekly test positivity of the occupational category or age group vs the reference group.

**Figure 2:**
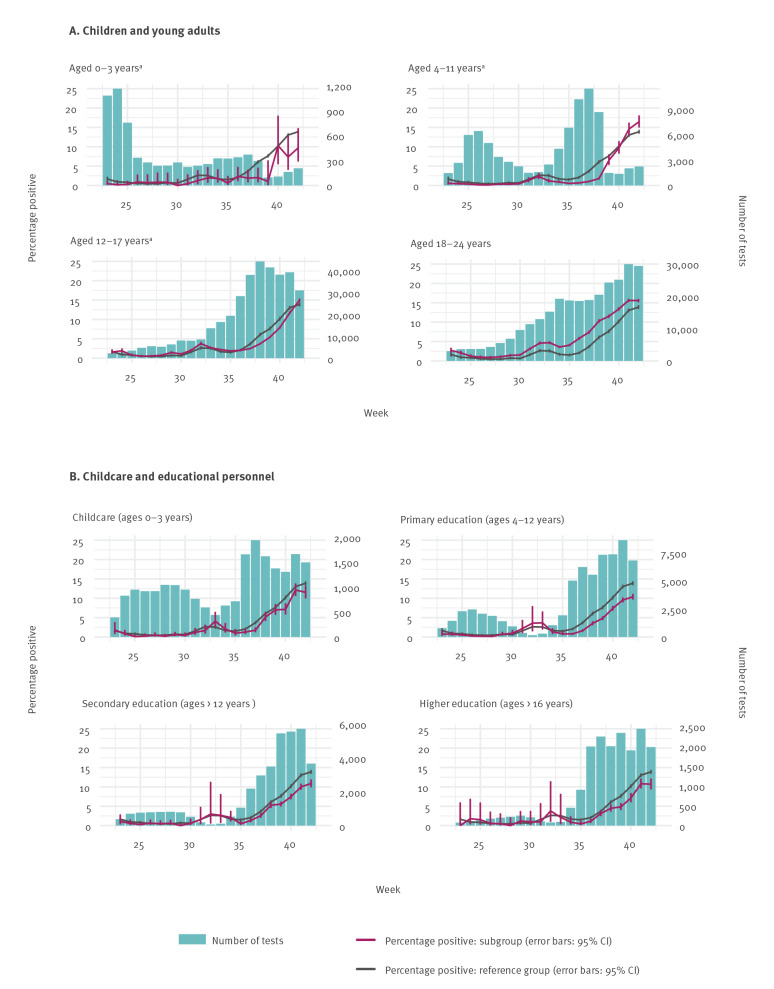
Number and percentages of SARS-CoV-2 PCR-positive (A) children and young adults and (B) childcare and educational personnel, the Netherlands, week 23–42 2020

CI: confidence interval; SARS-CoV-2: severe acute respiratory syndrome coronavirus 2.

^a^ From week 39 onward, children ≤ 12 years old with COVID-19 symptoms were no longer tested. Exceptions were severely ill children, symptomatic children who were a confirmed case contact or children who were part of an outbreak investigation.

**Figure 3:**
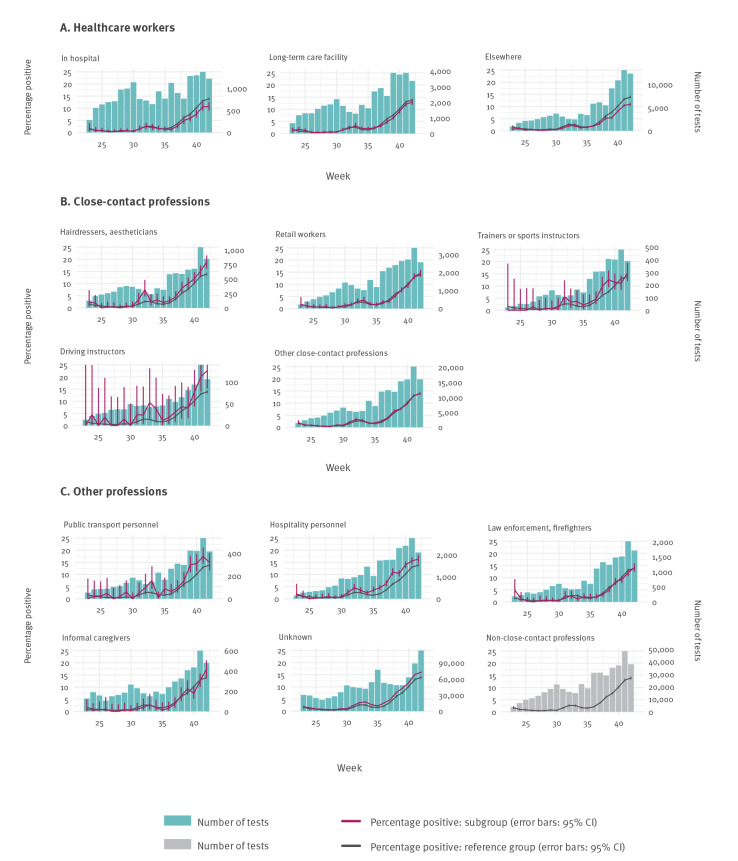
Number and percentages of SARS-CoV-2 PCR-positive (A) healthcare workers, (B) close-contact professionals and (C) other professionals, the Netherlands, week 23–42 2020

CI: confidence interval; SARS-CoV-2: severe acute respiratory syndrome coronavirus 2.

Figure 2 and Figure 3 show an overall increase in test positivity from 2 August (weeks 32–33), after which it dropped (weeks 34–35), and then steadily increased until the last week included in this analysis (week 42). The following occupational groups had generally higher test positivity than the reference group: people working in the hospitality and public transport sectors, driving instructors, hairdressers and aestheticians, although the differences in test positivity were not as pronounced in every week. Of note, the use of non-medical face masks has been obligatory in public transportation and advised for close-contact professions since 1 June; driving instructors were not advised to wear face masks until 19 October (week 43).

Please note the different scales of the right-hand y axes. 95% CIs are given as vertical error bars. Childcare and educational personnel subgroups reported working in these professions in the 2 weeks before testing. The black reference line in each subplot is the test positivity of adults that reported working in a non-close-contact profession in the 2 weeks before testing.

Please note the different scales of the right-hand y axes. 95% CIs are given as vertical error bars. Occupational subgroups reported working in these professions in the 2 weeks before testing. The black reference line in each subplot is the test positivity of adults that reported working in a non-close-contact profession in the 2 weeks before testing. Weekly numbers of tests for this reference group are shown in the last plot (grey bars).

Figure 2 and Figure 3 also show that during the second increase in test positivity, starting at week 36, educational personnel and HCWs showed lower test-positive percentages than the reference group. These results may indicate that these occupational groups complied with the precautionary measures, including proper personal protective equipment use among HCWs, where indicated. These results may also—at least in part—be caused by a higher testing rate among these professional categories, who—in general—cannot work from home.

Up to week 39, children < 12 years of age showed consistently lower test positivity percentages than adults. Young children are more likely to develop mild or no symptoms after SARS-CoV-2 infection [2]. Our data showed that among young children with symptoms (including mild symptoms), the SARS-CoV-2 test positivity was also lower. Since week 39, children < 13 years of age who had mild symptoms and no known contact with a laboratory-confirmed COVID-19 case were no longer advised to undergo SARS-CoV-2 testing because of limited testing capacity. Therefore, there was a reduction in testing among children in the age groups 0–3 years and 4–11 years (Figure 1), while this change in testing policy likely increased test positivity in these age groups. The lower test positivity among educational personnel compared with the reference group was in line with the lower test positivity among primary school students (up to week 39), supporting the hypothesis that transmission of SARS-CoV-2 by primary school children is likely modest, as has been described before [3]. Preventive measures have been installed in schools, such as physical distancing between teachers and students, and secondary school students have used non-medical face masks between classes since week 42 (Table 2).

**Table 2:** Selected COVID-19 prevention measures and testing policies, the Netherlands, 1 June–17 October 2020

Week	Prevention measures	Testing policies
**23**	Maintain physical distance of 1.5 m (> 12 years old) Non-medical face masks obligatory on public transport (> 12 years old) Cinemas, restaurants, cafes and cultural institutions (such as concert halls and theatres) allowed to open with precautionary measures and a maximum of 30 visitors Secondary schools are partly open; measures taken to prevent contact between students and teachers within 1.5 m	Individuals can schedule a SARS-CoV-2 PCR test if they have symptoms consistent with COVID-19^a^
**24**	Primary schools reopen 100% (were reopened 50% since 11 May 2020)	No changes
**27**	Students in secondary schools no longer have to keep 1.5 m distance from each other Communal sanitation facilities at campsites and holiday parks re-opened Maximum number of visitors expanded to 100 people for cinemas, restaurants, cafes and cultural institutions Fitness clubs, saunas, wellness centres, club canteens and casinos re-opened	No changes
**32**	Hospitality sector opened for reservations (in advance or at the door),with a health check and the allocation of a fixed seating placement at a table or at the bar (all visitors asked to register to enable contact tracing)	No changes
**34**	Gatherings in private homes limited to six people (residents and guests, aged > 12 years ) Individuals encouraged to work from home as much as possible Home quarantine reduced from 14 to 10 days	No changes
**39**	In regions with high incidence of SARS-CoV-2 infections: Bars and restaurants closed at 1:00 Maximum group size is 50 people	Due to shortages in testing capacities, children ≤ 12 years old with mild COVID-19 symptoms are no longer tested^b^ Essential healthcare and educational personnel can get tested with priority
**40**	Individuals to work from home as much as possible Gatherings in private homes limited to three guests, excluding children ≤ 12 years old Maximum group size elsewhere is four people > 12 years old, for instance for reservations in cinemas or restaurants The total number of people indoors is limited to 30 (> 12 years old), outdoors 40 Food and beverage outlets closed by 21:00, stores closed by 22:00 Canteens at sporting facilities closed, no spectators allowed at sporting events People with a close-contact profession must ask their customers to register Travel and movements limited as much as possible All people ≥ 13 years old urgently advised to wear a non-medical face mask in publicly accessible indoor areas	No changes
**42**	Partial lockdown Gatherings in private homes limited to three guests per day, excluding children≤ 12 years old In secondary and higher education, everyone must wear a mask outside of class All restaurants and bars closed (take-out is allowed) Public transport only for essential travel Retail stores closed by 20:00 No alcohol sold between 20:00 and 7:00 Public events cancelled For everyone ≥ 18 years old, sports only allowed at a distance of 1.5 m; maximum team size is four people At sporting facilities, canteens, showers and changing rooms are closed Travel abroad limited as much as possible	No changes

COVID-19: coronavirus disease; SARS-CoV-2: severe acute respiratory syndrome coronavirus 2.

^a^ Such as runny nose, sneezing, sore throat, cough, fever or sudden loss of smell or taste.

^b^ Exceptions are critically ill children, children who are a confirmed COVID-19 case contact or children who are included in an outbreak investigation.

The following prevention measures have been advised since 12 March and throughout the study period: frequent handwashing, sneeze and cough hygiene, physical distancing of at least 1.5 m, self-isolating when experiencing COVID-19-like symptoms or when a household member has a fever and/or shortness of breath, and avoiding crowded places.

## Discussion and conclusions

Evidence on occupational risk of testing positive for COVID-19 is thus far scarce. Our study’s finding of increased test positivity among workers in the hospitality and public transport sectors, driving instructors, hairdressers and aestheticians not only has implications for these professionals, but also for everyone who is using these services. The registration of occupation is therefore relevant for public health policymaking. Such data are useful to evaluate COVID-19 mitigation policies and identify sectors that may be eligible for more lenient restrictions or that may require stricter restrictions. Changes to testing practices in the near future, such as rapid antigen testing, should be embedded in this surveillance so that subgroup-specific surveillance of test positivity remains possible.

The data presented in this rapid communication have several limitations. Registration of profession was incomplete; in total, profession was ‘unknown’ for 36% (966,025/2,700,563) of tests (Supplementary Table S1). Completeness of information, however, improved over time: in week 23, the occupational category was unknown for 63% (30,375/47,803) of tests, vs 32% (88,771/276,362) of tests in week 41. The category ‘unknown’ includes individuals who did not work in the 2 weeks preceding their test—e.g. because of (full-time) education, unemployment, illness, vacation or retirement—and is therefore not solely ‘missing data’. Also, certain occupations with a high SARS-CoV-2 infection risk, such as food processing [4], could not be identified in our dataset, given the limited number of occupational categories in the questionnaire.

Test positivity might not translate to COVID-19 incidences equally for all subgroups. Important unknowns are the incidence of testing by group, the incidence of COVID-19–like symptoms by group and the testing behaviour by group. The number of tests per 100,000 population, by age group, can be determined based on demographic data. Denominators for the different specific occupational groups are much more uncertain. Furthermore, since patients are categorised in an occupational group in CoronIT only if they have reported active work in the 2 weeks before their test—and because this question regarding occupation is not always filled in—the numbers cannot simply be divided by denominators of known occupational group sizes. Test behaviour is also likely to differ between occupational groups, e.g. because of differences in risk perception and the extent to which it is possible to work from home. For example, for small business owners in the hospitality sector, the threshold for testing may be relatively high. In October, there were some weeks of test scarcity, during which people may have been discouraged from testing due to long delays between applying for a test and receiving results (90 hours on average in week 41), during which time patients were asked to self-isolate. As of 21 September (week 39), essential HCWs and educational personnel received priority testing to facilitate their swift return to work; since this date, the number of tested individuals among HCWs and educational personnel (especially those working in secondary education) increased and the curves for HCWs (in hospital and elsewhere) and educational personnel (secondary and higher) diverged more from the reference group.

The incidence of symptoms in different groups is even more uncertain. A novel population-based syndromic surveillance system, named Infectieradar and modelled on influenzanet [5], was recently implemented to provide more insight into the incidence of COVID-19–like symptoms. It may be hypothesised that occupational groups such as HCWs, childcare personnel and educational personnel might be at an increased risk of exposure to other respiratory pathogens with symptoms similar to COVID-19, such as rhinovirus, rendering these professionals more likely to get tested and to test negative for SARS-CoV-2.

Published data on COVID-19–associated deaths [6,7] and hospitalisations [8] by occupation have highlighted HCWs as being at increased risk for severe COVID-19, which is in contrast to our finding of lower test positivity in this group. It is possible that there is selection bias in our data on HCWs, as HCWs employed by a hospital or long-term care facility with direct links to a hospital can be tested via their employer (the hospital laboratory) rather than the PHS test locations. These tests from outside of the PHS are not registered in the CoronIT database and are therefore not included in our analysis. However, some hospital laboratories report the numbers of negative and positive tests directly to RIVM, and separately for patients and employees. In these separate data, the percentages positive among hospital employees are slightly lower than in the tested persons registered in CoronIT as ‘HCW in hospital’ [9]. The risks of SARS-CoV-2 infection and severity for HCWs do not appear to be higher than average, based on notifications received between June and October (data not shown). Compared with other notified cases aged 17–69 years, we have seen relatively low percentages of hospitalised (0.3% vs 0.7% of non-HCW notified cases) and deceased HCWs (0.01% vs 0.05%) since 1 June. For the first wave of COVID-19 in the Netherlands, such comparisons are less informative due to testing policy prioritising HCWs.

### Conclusions

Despite its limitations, the system CoronIT provides the unique possibility of detailed nationwide surveillance of SARS-CoV-2 by population subgroup in the Netherlands. This source of surveillance data will allow evaluation of the COVID-19 epidemic’s development in specific subpopulations and occupational groups targeted by prevention measures. We believe these population-level, subgroup-specific surveillance data can also be informative for European countries that do not have data on specific target groups and may inform choices in national policies to slow the spread of COVID-19.

## Supplementary Data

427000 Supplementary Table S1
