# Supplementary material for: Occupation- and age-associated risk of SARS-CoV-2 test positivity, the Netherlands, June to October 2020
Source: Euro Surveill. 2020 Dec 17;25(50):2001884. doi: 10.2807/1560-7917.ES.2020.25.50.2001884 (PMC7812419; doi:10.2807/1560-7917.ES.2020.25.50.2001884)
Supplement: Supplementary Table S1 [file 2001884_SuppTable1.pdf]

This supplementary material is hosted by *Eurosurveillance* as supporting information alongside the article Occupation- and age-associated risk of SARS-CoV-2 test positivity, the Netherlands, June to October 2020, on behalf of the authors, who remain responsible for the accuracy and appropriateness of the content. The same standards for ethics, copyright, attributions and permissions as for the article apply. Supplements are not edited by *Eurosurveillance* and the journal is not responsible for the maintenance of any links or email addresses provided therein.

Supplementary Table S1. Total number of SARS-CoV-2 PCR tests and number positive, per age or occupational category, the Netherlands, week 23-42 2020

Category	Number tested	Number positive
**Children aged 0-3**	7292	100
**Children aged 4-11**	83826	1475
**Children aged 12-17**	364419	19604
**Young adults aged 18-24**	283138	23586
**Childcare personnel (ages 0-3)**	21187	790
**Primary education personnel (ages 4-12)**	63954	2877
**Secondary education personnel (ages 12+)**	36533	2026
**Higher education personnel (ages 16+)**	17850	928
**HCW in hospital**	17747	635
**HCW in long-term care facility**	42656	2039
**HCW elsewhere**	97260	4957
**Close-contact profession: Hairdressers, estheticians**	8669	626
**Close-contact profession: Retail workers**	30232	1723
**Close-contact profession: Trainer or sports instructors**	3794	285
**Close-contact profession: Driving instructors**	1073	96
**Close-contact profession: Other**	166726	10157
**Public transit personnel**	4343	357
**Hospitality personnel**	23910	1946
**Law enforcement, firefighters**	15852	948
**Informal caregiver**	5045	280
**Other non-close-contact professions**	439032	23977
**Unknown**	966025	61099
